# Voice recognition as an innovative method to identify and monitor heart failure – a review

**DOI:** 10.1007/s10741-026-10625-3

**Published:** 2026-04-16

**Authors:** Amy S. Fuller, Nduka C Okwose, Sarah J. Charman, Alban E. Voppel, Renae J. Stefanetti, Amy Groenewegen, Annamaria Del Franco, Maria Tafelmeier, Andrej Preveden, Aleksandra Milovancev, Duncan Edwards, Anne P. Nelissen, Fausto Barlocco, Alessandra Fornaro, Jose Luis Zamorano, Massimo Milli, Laura Sasso, Giulia Taborchi, Simone Bartolini, Serena Fatucchi, Prithwish Banerjee, Guy A. MacGowan, Oscar Fernandez, Marta Jimenez-Blanco Bravo, Lars S. Maier, Iacopo Olivotto, Frans H. Rutten, Jonathan Mant, Lazar Velicki, Petar M. Seferovic, Nenad Filipovic, Djordje G. Jakovljevic

**Affiliations:** 1 Clinical Sciences and Translational Medicine Research Theme, Research Centre for Discoveries in Life Sciences, Coventry University, Coventry, UK; 2 University Hospitals Coventry and Warwickshire NHS Trust, Coventry, UK; 3 Translational and Clinical Research Institute, Faculty of Medical Sciences, Newcastle University, Newcastle upon Tyne, UK; 4 Newcastle upon Tyne Hospitals NHS Foundation Trust, Newcastle upon Tyne, UK; 5 Centre of Excellence in Youth Mental Health, Douglas Mental Health University Institute, McGill University, Montreal, Canada; 6 National Institute for Health and Care Research (NIHR) Newcastle Biomedical Research Centre (BRC), Newcastle upon Tyne, UK; 7 Department of General Practice & Nursing Science, Julius Centre for Health Sciences and Primary Care, University Medical Centre Utrecht, Utrecht University, Utrecht, Netherlands; 8 Careggi University Hospital, University of Florence, Florence, Italy; 9 Department of Internal Medicine II, University Medical Centre Regensburg, Regensburg, Germany; 10 Faculty of Medicine, University of Novi Sad, Novi Sad, Serbia; 11 Institute of Cardiovascular Diseases Vojvodina, Sremska Kamenica, Serbia; 12 Primary Care Unit, Department of Public Health and Primary Care, University of Cambridge, Cambridge, UK; 13 Biosciences Institute, Newcastle University, Newcastle upon Tyne, UK; 14 Hospital Universitario Ramón y Cajal, Madrid, Spain; 15 Centro de Investigación Biomédica en Red CIBER-CV, Instituto de Salud Carlos III, Madrid, Spain; 16 Cardiology and Electrophysiology Unit, Department of Medical Specialties, Azienda USL Toscana Centro, Santa Maria Nuova Hospital, Florence, Italy; 17 Department of Cardiology, Faculty of Medicine, University of Belgrade, Serbian Academy of Sciences and Arts, Heart Failure Society of Serbia, Belgrade, Serbia; 18 Bioengineering Research and Development Center, BioIRC, Kragujevac, Serbia; 19 Faculty of Mechanical Engineering, University of Kragujevac, Kragujevac, Serbia

**Keywords:** Heart failure, Vocal biomarkers, Novel technique, Screening, Risk stratification

## Abstract

Screening of heart failure (HF) remains suboptimal. However, vocal biomarkers are an emerging tool to screen for HF and predict adverse outcomes. This article is a literature review of the current evidence, exploring the use of vocal biomarkers to screen for HF and predict hospitalisation and mortality. Voice may be utilised as a time-efficient method for the screening of HF, risk stratification, and monitoring of disease progression. Vocal biomarkers such as pause ratio and artificial neural networks can screen for HF accurately in a case-control setting. Other vocal markers like maximum phonation time, creak percent are useful to monitor disease progression. Vocal biomarkers appear to match current predictors of one-year mortality, HF related decompensation and hospitalisation. The integration of vocal biomarkers assessment in HF healthcare is promising across primary and secondary care settings. Key challenges including health data protection, regulatory compliance, and user acceptance must be addressed before these tools can be fully integrated into HF clinical care pathway.

## Abstract (highlights)

**Key messages:**

*****What is known***:**

- Heart failure (HF) remains under-diagnosed, particularly in early stages and community settings, despite guideline-directed diagnostic tools.

- Voice features and machine-learning–derived vocal biomarkers differ between individuals with HF compared to healthy controls and may be associated with hospitalisation and mortality, but studies are currently limited.

*****What this review adds***:**

- Synthesised evidence supporting voice-based biomarkers as a non-invasive diagnostic approach for heart failure screening, risk stratification, and diagnosis.

- A physiological framework has been provided linking HF pathophysiology to measurable voice changes and highlights the potential of voice analysis for scalable telemedicine, while addressing key methodological, ethical, and regulatory challenges.

**Graphical Abstract:**

## Graphical abstract

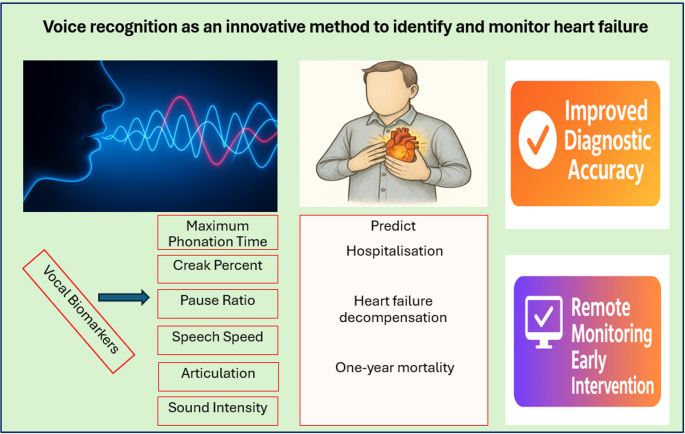

## Introduction

Heart failure (HF) is an increasingly prevalent and complex clinical syndrome that affects ~ 64 million (1–3%) of the global population and ~ 15 million individuals (1–2%) in Europe [1, 2]. If unrecognised HF was considered, such prevalence numbers would double [3, 4].

Despite the advancement in diagnostic techniques, screening and risk stratification for HF remains suboptimal. Analysis of vocal features and biomarkers has been increasingly recognised as having screening and risk-stratification potential for diseases affecting organs such as the heart, lungs, and brain. Human voice production is a complex biomechanical process that depends on the coordinated interaction of the respiratory system, laryngeal structures, and subpharyngeal vocal tract [5, 6]. At its core, phonation occurs when airflow generated from the lungs, passes through the larynx and induces vibration of the vocal folds. These vibrations generate an acoustic signal that is subsequently shaped by the resonant properties of the pharynx, oral cavity, and nasal passages. Production of voice and its features is complex, and attributed to interaction between the larynx, articulators such as the tongue, lungs, and the autonomic nervous system [7]. A glossary of vocal metrics has been provided in Table 1.

**Table 1:** Voice-based biomarkers

Biomarker	Definition	How it is measured	Physiological basis
Maximum phonation time (MPT)	The maximum duration (in seconds) that an individual can sustain a vowel sound (commonly/a/) on a single breath at comfortable pitch and loudness.	Patient sustains a vowel after maximal inhalation; longest duration from repeated trials is recorded.	Reflects integrated respiratory capacity, airflow control, and vocal fold efficiency. Requires stable subglottic pressure and coordinated respiratory–laryngeal function.
Creak percent	The proportion of speech produced using creaky voice (low-frequency, irregular vocal fold vibration) relative to total voiced speech.	Acoustic analysis of connected speech to quantify irregular, low-frequency phonation patterns.	Creaky phonation arises from altered vocal fold tension, reduced airflow, or laryngeal compensatory strategies under physiological stress.
Pause ratio	The proportion of total speaking time spent in silence or pauses.	Calculated as total pause duration divided by total speech duration during a speaking task.	Pausing reflects breathlessness, reduced respiratory endurance, and impaired speech–breathing coordination.
Speech speed	The rate of speech production, typically measured as syllables or words per second.	Derived from time-aligned speech recordings during reading or spontaneous speech tasks.	Dependent on respiratory support, neuromuscular coordination, and cognitive-motor integration.
Articulation	The clarity and precision with which speech sounds are produced.	Assessed via acoustic measures (e.g. formant transitions) or perceptual analysis of speech clarity.	Requires coordinated movement of the tongue, lips, jaw, and soft palate, supported by adequate airflow and neuromuscular control.
Sound intensity (Loudness)	The acoustic energy of the voice signal, typically measured in decibels (dB).	Measured using calibrated microphones or relative intensity metrics from recordings.	Sound intensity depends on subglottic pressure and effective vocal fold closure.

Reproduced based on works of Titze IR. *Principles of voice production.* Prentice Hall; 1994.

Sataloff RT. *Clinical assessment of voice.* Plural Publishing; 2017

Kent RD, Read C. *The acoustic analysis of speech.* Singular Publishing Group; 2002

Voice recording to identify vocal biomarkers has no gold standard protocol in cardiovascular clinical practice. However, guidelines for the recording and utilisation of voice are provided by phoniatric professionals such as the European Laryngological Society and American Speech-Language-Hearing Association [8, 9]. Voice is typically assessed utilising tasks that address the following 3 categories: (i) isolated words, short sentence repetitions, reading a passage and running speech for verbal features; (ii) sustained vowel phonation and diadochokinetic tasks for vowels/syllables; and (iii) coughing and breathing are assessed for nonverbal vocalisation [5]. Techniques for data collection can be studio-based, telephone-based, web-based, or smartphone-based. Analysis of voice features and vocal biomarkers in clinical research utilise typical statistical and machine learning methods [10]. Once extracted, data may be used to train machine learning models which must be validated and examined for their prognostic potential.

Inspiration for developing speech-analysis systems in HF emerged from informal observations that individuals with HF could exhibit distinct speech patterns either in congested (wet) or normal (dry) states [11].

Despite evidence of altered voice features in individuals with HF, the underlying pathophysiological mechanisms remain incompletely understood. To add further complexity, most individuals with HF are older adults who, in addition to fluid overload, often exhibit age-related vocal-cord atrophy and reduced elasticity [12]. Such alterations are aetiological of presentations such as resistance to vocalisation, difficulty reproducing high pitches, and hoarseness [12]. However, potential pathophysiological mechanisms for voice feature alterations in HF include dysregulation of the autonomic nervous system, fluid retention, and hydrostatic pressure within the pulmonary system.

Dysregulation of the autonomic nervous system is well documented in individuals with HF and contributes to haemodynamic abnormalities [13]. This manifests as increased sympathetic activity and decreased vagal tone. Consequently, it is plausible that these autonomic changes in HF may result in measurable alterations in vocal characteristics [7, 14].

A key sign and symptom of congestive HF is fluid retention (oedema), which can occur across a wide range of tissues [15]. Progression of pulmonary oedema is the primary cause of hospitalisation and is related to adverse outcomes post-hospitalisation in individuals with acute decompensated HF (Boorsma et al., [16]). Therefore, monitoring fluid status by measuring body weight at the same time each day is an essential component of the HF clinical care pathway [17]. However, changes in body weight typically occur only after substantial fluid accumulation, limiting the ability of weight-based assessments to predict or provide early warning of impending fluid overload. Clinically relevant changes in vocal biomarkers have been reported to confirm decompensation after much smaller fluid changes in the larynx [15, 18, 19]. Furthermore, in individuals with congestive HF, increased hydrostatic pressure results from inadequate pumping of the heart, leading to fluid leaking from capillaries into the pleural space (i.e., pleural effusion) and affecting the lung component of voice production [20]. In coherence with the described changes, biomarkers to confirm pulmonary oedema in individuals with HF when compared to healthy controls have been reported to achieve high accuracy (i.e., specificity and sensitivity > 95%) [21]. Therefore, language-independent vocal biomarkers may offer an efficient and timely approach for detecting decompensation and adverse outcomes in individuals with heart failure, enabling earlier clinical intervention.

Developed artificial neural networks may be used to screen for HF through the generation of vocal biomarkers capable of identifying the pathological cause of non-specific cardiorespiratory symptoms such as shortness of breath (SOB) [22, 23]. These vocal biomarkers have also been suggested to be predict risk of mortality and hospitalisation in individuals with HF [14, 24].

The present article discusses vocal biomarkers and their potential to screen for HF [22, 23], predict risk of adverse outcome following diagnosis [14], and determine disease progression. Regarding HF progression, the association with maximum phonation time (MPT) [25–28], creak percentage [18, 29], and pause ratio [30] are explored. Overall, fourteen manuscripts are discussed in the present scoping review under the following themes.

## Heart failure screening and vocal biomarkers

There is growing evidence to suggest that vocal biomarkers may be utilised as adjunctive technology to facilitate the screening of HF in the future (Table 2). During the diagnostic evaluation of suspected HF based on history and signs and symptoms, the most recent European Society of Cardiology and National Institute for Health and Care Excellence (NICE) guidelines recommend measuring natriuretic peptides (NP) levels and electrocardiography (ECG) without delay, prior to echocardiography assessment [15, 32]. Despite these recommendations, NP testing remains underutilised prior to confirming HF, with fewer than 25% of individuals receiving such testing in primary care within six months preceding diagnosis [33]. This underutilisation may reflect limited access to equipment, time constraints, cost considerations, or the invasive nature of obtaining blood samples. The key advantages of using voice recordings for HF risk stratification and screening include its low cost, and greater scalability across the general population compared with traditional blood-based biomarkers and echocardiography. Unlike the acquisition of blood biomarker results, voice recording is non-invasive, rapid, requires minimal equipment, and can be performed immediately by the treating clinician when HF is suspected, without the delay associated with sending patients for blood tests. Artificial neural networks that analyse voice are currently being developed for HF screening [22, 23]. In completed studies (Table 2), voice data were collected in either Brazilian Portuguese [22] or Finnish [23] languages. Notably, the required recording tasks took less than one minute for clinicians to administer, and in some cases, voice samples were obtained from routine clinical phone calls. These findings underscore the global applicability and practical feasibility of voice‑based assessment in clinical settings. In the context of single vocal biomarkers for HF screening (Table 1), speech rate (the speed of talking) and cepstral peak prominence (CPP), defined as the difference in decibels between the highest cepstral peak and the noise floor, have shown potential diagnostic value. CPP can be evaluated specifically from vocal-fold vibration (CPP-V) signals or from all speech signals, including voiced and unvoiced phonemes and pauses. These biomarkers have been suggested to help discriminate between individuals with pulmonary hypertension or chronic stable HF and those without cardiopulmonary disease [31]. Pulmonary hypertension is highly prevalent in HF and may occur with or without systolic dysfunction [34, 35]. Sara et al. further demonstrated that CPP-V has the potential to distinguish between healthy individuals and those with pulmonary hypertension, a difference not observed among individuals with HF alone or those with combined disease phenotypes [31]. This finding is particularly noteworthy given the well-established association between pulmonary hypertension and increased morbidity, mortality, and reduced quality of life when it coexists with HF.

**Table 2:** Studies exploring vocal biomarkers for the screening of heart failure

Reference	Overview	Study *N* (% male)	Age, mean (range OR SD)	Results
[22]	**Measures assessed**: Two artificial neural networks specific to gender. Assessment of vocal distortions (utilising frequency ranges) to identify HF. **Recording technique**: PX440 voice recorder (Sony) was used to collect voices in a quiet hospital room. 20 cm between interviewee and recorder. Reading of a short sentence three times in native language (Portuguese) 3x.	H: 58 (50) HF: 84 (66)	Hm: 52 ± 12.5 Hfe: 53 ± 11.6 HFm: 56 ± 13.5 HFfe: 55 ± 10.9	Efficiency: 96.7% Accuracy: 91.9% Sensitivity: 88.1% Specificity: 92.1%
[23]	**Measures assessed**: Mel-frequency cepstral coefficient features, glottal features, and their combination. Four machine learning algorithms (support vector machine, Extra Tree, AdaBoost, and FFNN were separately trained for individual features and combination. **Recording technique**: Speech data sampled in Doctor’s practice rooms at 44.1 kHz, using a condenser microphone (DPA 4065-BL). Each speaker read same Finnish text (91 words) 3x and produced one spontaneous speech. Middle recitation was used for reading task. Linear phase FIRfilter (cut-off frequency at 60 Hz) used to remove low-frequency noise.	H: 25 (72) HF: 20 (70)	60 (54–83) 67 (36–81)	Superior FFNN accuracy- combined and specific: 95.02%
[31]	**Measures assessed**: Sentence extracted from a standardised text and analysed to extract acoustic features (i.e., including SR and CPP (CPP-V; CPP-A)) in PH, HF, combined or patients with no disease. **Recording technique**: iPad 3rd Generation, Apple 2012, 20 cm from mouth. Passage from ‘The Magic of Oz’ read. audio was normalized using a root mean square level set at 0.5. Speech signals sampled at 44.1 kHz with 16-bit quantization, and high‐pass filtered at 70 Hz to remove low‐frequency noise artefacts using Praat (version 4.07, 2024) **Analysis**: Praat software.	Overall: 2153 HF: 879 PH: 542 ND-: 777 PH-: 156 HF-: 396 PH + HF-: 218 PH+: 66 HF+: 163 PH + HF+: 102 ND+: 275	65.32 ± 17.2; 60.4 ± 14.2; 66.5 ± 13.3; 68.2 ± 12.9; 70.3 ± 15.3; 71.3 ± 13.1; 61.5 ± 12.2; 74.5 ± 12.0; 60.8 ± 15.8	SR slower in patients with HF, PH, and combined compared to absence of disease. CPP-V differed for PH, but not HF when compared to no disease.

Abbreviations:* CPP* cepstral peak prominence, *CPP-A* CPP-all speech, *CPP-V* CPP voiced speech, *FFNN* Feed-forward neural network, *H* healthy, *HF* heart failure, *Hfe* healthy female, *HFfe* Heart failure female, *Hm* healthy male, *HFm* Heart failure male, *ND* No disease, *PH* pulmonary hypertension, *SD* standard deviation, *SR* speech rate

Annotations: -: absence of symptoms; +: presence of symptoms

### Heart failure adverse outcomes and vocal biomarkers

Research suggests that vocal biomarkers can predict the risk of adverse outcomes such as mortality and hospitalisation in individuals diagnosed with HF (Table 3). Risk predictors defined by [36] for HF all-cause mortality include age, New York Heart Association (NYHA) functional class, left ventricular systolic dysfunction, atrial septal defect, aortic valve annulus calcification, tricuspid regurgitation severity, QRS duration, T wave offset, and NT-proBNP. Such risk factors are demonstrated good at discrimination of 2-, 3-, and 5-year mortality in training (i.e., area under curve (AUC)s: 0.73, 0.76, 0.81, respectively) and validation cohorts (i.e., AUCs: 0.69, 0.68, 0.71) [36]. In contrast, the prediction of mortality in hospitalised patients using contemporary models is only moderately successful (i.e., AUC: 0.66). The ability to identify high-risk patients in a timely manner is imperative for early optimisation of treatment strategy.

**Table 3:** Studies exploring vocal biomarkers for heart failure outcomes

Reference	Measures assessed	Study *N* (% male)	Age, mean (range OR SD)	Results
​​(Maor et al., 2020)​ [14]	**Measures assessed**: Association of vocal signal analysis with adverse outcome in individuals with CHF. 223 acoustic features extracted from 20 s speech from a phone call. Risk of death and hospitalisation assessed. Evaluation as a continuous and ordinal (4 quartile) variable. **Recording technique**: Nurse patient centre’s protocol call recorded. At least 20 s recording utilised. Specific voice elements, not verbal recording utilised for analysis. Most recent call used.	CHF: 2267 (63)	77: (68–83)	Independent association of VB with hospitalisation. Increased cumulative probability of death with increasing quartile- 23%, 29%, 38%, 54%. 32% increased risk of death with each SD increase in VB during follow-up (mean 20mo).

*CHF* Congestive heart failure, *SD* standard deviation, *VB* vocal biomarker

Maor and colleagues developed a vocal biomarker identified by a machine learning algorithm incorporating 223 acoustic features recorded in Hebrew or Russian [14]. In this study, a training dataset of non-chronic HF recordings were used to develop a biomarker, which were then tested on a group of individuals with the disease. The vocal biomarker was observed to have moderate success in predicting risk of 1-year mortality in HF (i.e., all-cause mortality AUC of 0.67), like the presently utilised risk factor models in individuals with congestive HF [14]. Where the vocal biomarker was detected, individuals were observed to have an approximately one-third increased risk of mortality at follow-up (i.e., 20 months). Poor survival persisted even after adjusting for confounding factors including age, sex, and additional clinical and laboratory predictors of death such as hypertension and triglycerides. The study also assessed the ability of the vocal biomarker to determine risk in four quartiles, with the highest quartile showing a 96% increased likelihood of death compared to lowest quartile [14]. Therefore, the identified vocal biomarker may be an efficient and non-invasive solution to identify individuals with HF at risk of adverse outcome.

## Heart failure progression and vocal biomarkers

The pathway for HF management requires improvement. Development of novel methods will lead to more timely intervention to manage disease progression. Several approaches for the remote, long-term management of HF have been explored with the objective of identifying and intervening upon clinical decompensation at an earlier stage. These include B‑type natriuretic peptide (BNP) guided therapy and remote haemodynamic monitoring with clinical reporting [37–39]. However, evidence supporting these approaches has been inconsistent, and their implementation often necessitates invasive procedures, such as repeated blood sampling or the implantation of monitoring devices (e.g. pressure sensors.

Based on current literature (Table 4), vocal biomarkers display potential to mirror and/or outperform traditional markers of exercise capacity, mortality, and HF congestion. With Amir and colleagues confirming over 90% accuracy of machine learning to compare voice features between treated and hospitalised individuals with HF [11]. Pathophysiological progression in HF including altered vagus nerve functioning, pulmonary oedema, and reduced laryngeal perfusion and compression of the left laryngeal nerve due to fluid retention are all suggested to contribute to changes in voice over time [13, 40].

**Table 4:** Studies exploring heart failure progression and vocal biomarkers

Reference	Measures assessed	Study N (% male)		Age,mean (range OR ± SD)	Results			
MPT								
​​(Izawa, Watanabe, Tochimoto, et al., 2012)​ [25]	**Measures assessed**: Relationship between MPT and exercise capacity; MPT needed for an exercise capacity ≥ 5 METs in CHF outpatients. **Recording technique**: MPT measured with a stopwatch (s). Patients asked to produce sustained vowel/a:/for as long as possible.	CHF: 111 (ND)		54.2 ± 10.1	Higher MPT in CHF ≥ 5 MET. MPT cut-off value 18.27s Sens: 0.76 Spec: 0.33 AUC: 0.81			
​​(Izawa, Watanabe, Brubaker, et al., 2014)​ [28]	**Measures assessed**: Relationship between the VE/VCO_2_ slope and MPT. MPT required for a VE/VCO_2_ slope value of ≤ 34 in CHF individuals. **Recording technique**: MPT measured with a stopwatch (s). Patients asked to produce sustained vowel/a:/for as long as possible. 3x trials complete with a 15 s break between each. Highest value measured was considered the index of MPT (s).	CHF: 115 (85) Slope ≤ 34: 81 (85) Slope > 34: 34 (84)		54.5 51.5 ± 8.9 61.3 ± 9.6	Significantly negative correlation between V̇E/V̇CO_2_ slope and MPT. MPT cut-off of ≤ 34 V̇E/V̇CO_2_ slope 18.12s			
​​(Izawa, Watanabe, Tochimoto, et al., 2014)​ [26]	**Measures assessed**: The relationship between MPT index and disease severity in CHF males. **Recording technique**: *refer to ​​(Izawa, Watanabe, Brubaker, et al., 2014)​	CHF: 101 (100) NYHA I: 28 NYHA II: 49 NYHA III: 24		54.5 48.9 ± 8.4 56.1 ± 9.3 56.0 ± 11.7	Peak V̇O_2_ and MPT decrease significantly with increasing NYHA class.			
​​(Izawa, Watanabe, Oka, Tochimoto, et al., 2015)​ [27]	**Measures assessed**: Longitudinal changes in MPT and Peak VO_2_ after a 5-month home-based exercise training and MP exercise intervention; the relation between delta MPT and delta peak VO_2_ in CHF individuals. **Recording technique**: *refer to ​​(Izawa, Watanabe, Brubaker, et al., 2014)​	CHF: 36 (78)		55.1 ± 12.5	MPT and peak V̇O_2_ increased after 5 months. Delta MPT correlated positively with delta peak V̇O_2_.			
​​(Murton et al., 2023)​ [29]	**Measures assessed**: Acoustic measures of vocal perturbation and speech breathing characteristics from sustained vowels and speech passages recorded daily, including day of enrollment in ADHF individuals receiving diuretic treatment until discharge (Maximum 10 recordings). Measures assessed included (i) MPT (ii) rainbow passage (iii) second reading passage (iv) 30 s spontaneous speech. **Recording technique**: (H1 Handy Recorder, Zoom Corporation, Tokyo, Japan) and neck-mounted accelerometer (BU-27135; Knowles Corp., Itasca, IL). The microphone signal was sampled at 44.1 kHz and 16-bit quantization. All acoustic recordings were high-pass filtered at 70 Hz.	ADHF: 52 (62)		72 (34–96)	Increased MPT Acoustic speech measures and ability to distinguish pre-pose-treatment speech samples: Accuracy: 69% Sensitivity: 71% Specificity: 67% Best predictors for post-treatment: higher MPT, phonation stability, increased creak percent, faster speech rate, reduced pausing.			
​​(Okada et al., 2024)​ [24]	**Measures assessed**: (i) sustained a vowel (/a/) for as long as possible; and (ii) repeated the trisyllable (/pataka/) 5 times or more as fast as they could. 27 features within both in total. **Analysis**: ML models were created using acoustic features utilising a Light Gradient Boosting Machine. To confirm that AI methods would work with the present study’s sample size, ML models were also created and tested by a support vector machine. Shapley Additive exPlanations values were calculated to interpret the influence of individual acoustic features on HF conditions and improve explainability of the models. **Recording technique**: directional pin microphone connected to a portable, linear pulse-code modulation recorder at a sampling rate of 192 kHz with 24-bit resolution.		AHF: 59 (58)	74: (63–81)	27 acoustic features correlated with conventional HF indicators. S_DUR_01 (sustained vowel sound) differed between NYHA class ≥ 2 and < 2.			
Creak percent								
​​(Murton et al., 2017)​ [18]	**Measures assessed**: Acoustic measures of vocal perturbation and speech breathing characteristics from sustained vowels and speech passages recorded daily including (i) sustained vowels; (ii) CAPE-V sentences; (iii) the rainbow passage; (iv) spontaneous speech in ADHF individuals receiving diuretic treatment of decompensation. **Recording technique**: *refer to (Murton et al., 2023).	HF: 10 (80)		70 ± 13	Increased creak percent and decreased CPP SD of sustained-vowel utterance after treatment.			
Pause ratio								
​​(Schöbi et al., 2022)​ [30]	**Measures assessed**: Five tasks (i.e., reading patient consent; ascending number counting test; reverse number counting test; Stroop colour word reading test; Stroop colour word reading with randomised word-ink combinations test). Speech and pause patterns were extracted and compared between AHF and stable CHF individuals (i.e., rate of speech timing, pause ratio, mean pause duration, mean articulation duration) **Recording technique**: Web-browser based application, which included the tasks described.	AHF: 68 (72); Stable CHF: 36 (89)		76.1 ± 9.2 67.2 ± 12.8	Increased pause ratio in AHF compared to stable CHF individuals.			
Speech speed, articulation, and sound intensity								
​​(Schöbi et al., 2022)​ [30]	*Refer to ‘Pause ratio’	AHF: 68 (72); Stable CHF: 36 (89)		76.1 ± 9.2 67.2 ± 12.8	Abnormal lung auscultation in AHF.			
​​(Okada et al., 2024)​ [24]	*Refer to ‘MPT’	ADHF: 52 (62)		72 (34–96)	Correlation between I_F01 (one of the indices related to sound intensity) and BNP.			

*ADHF* acute decompensated heart failure, *AHF* acute heart failure, *AI* artificial intelligence, *BNP* B-type natriuretic peptide, *CAPE-V* consensus auditory-perceptual evaluation of voice, *CPP* Cepstral peak prominence,* CHF* Chronic heart failure, *H* healthy, *ML* machine learning, *MPT* Maximum phonation time, *METs* Metabolic equivalents, *ND* Not Defined, *V̇O*_*2*_ carbon dioxide production, *VE* ventilation, *V̇O*_*2*_ oxygen consumption, *POMV* Pulmonary oedema mechanical ventilation, *REPO* radiographically evident pulmonary oedema, *s* seconds, *SD* standard deviation, *SM* speech measure

AHF: 59 (58)

74: (63–81)

### Maximum phonation time

Maximum phonation time (MPT) assesses phonatory mechanics and has shown promise in the prediction of disease severity and prognosis in chronic HF. Such measure has been shown to be a highly reliable measure of voice assessment [41]. In contrast to key HF indicators measured at hospital discharge, including B-type natriuretic peptide (BNP), NYHA Functional class, and LVEF, voice features during MPT are observed to be significantly different in individuals with worsening HF at discharge than those without [24]. Similarly, MPT in individuals with acute decompensated HF significantly differed between NYHA Functional classes ≥ 2 and < 2, with measurements over a threshold of 10.62 s being associated with improved LVEF [24]. These findings suggest that language-independent components of voice may be utilised for decompensation risk.

Maximum phonation time (MPT) has also been shown to predict exercise capacity, as measured by peak oxygen uptake (V̇O_2_peak), and ventilatory efficiency, quantified using the ventilation efficiency slope (VE/V̇CO_2_), in individuals with HF [25, 26, 28]. V̇O2peak and the VE/V̇CO_2_ slope are typically assessed using cardiopulmonary exercise testing (CPET) and, while these parameters may also be influenced by other chronic conditions, they independently predict prognosis in individuals with HF [42]. Despite the established utility of CPET in identifying exercise intolerance and stratifying clinical risk, its use is limited by the need for specialised equipment, experienced personnel, and a controlled laboratory environment to ensure patient safety. Furthermore, a substantial proportion of individuals within the HF population have contraindications to CPET, restricting its applicability in routine clinical practice [43]. Therefore, the ability of MPT to predict V̇O_2_peak and VE/VCO_2_ slope without the need for a CPET has the potential to enhance clinical assessment and management for a greater proportion of individuals with HF [44]. Particularly as exercise intolerance is a key symptom of HF [15].

Lower MPT is associated with reduced respiratory function and higher NYHA in individuals with chronic HF [26]. Furthermore, lower MPT in individuals with HF is a predictor of reduced VO_2_peak, with a threshold for significant differences of 5 metabolic equivalents (METs) and negatively correlated with the VE/VCO_2_ slope [25]. A VE/VCO_2_ of 34 is significantly and independently predictive of mortality, and therefore disease progression, when exceeded in individuals with chronic HF [28]. Relevant to the described clinical outcomes, an MPT threshold measurement of 18.12 s is associated with such measurement.

Research on MPT and HF deterioration may also aid the development of novel therapeutic interventions. For example, it has been suggested that implementing home-based aerobic exercise (i.e., walking) and resistance exercises (i.e., body weight resistance training) in combination with maximum phonation exercises improve both exercise capacity and MPT [27]. The maximum phonation exercise involved sustaining a vowel sound (/a:/) for as long as possible, one, two and/or 3 times per week for five months, in addition to aerobic and resistance exercise [27]. Although conducted in a small study cohort (*n* = 36), this research suggests that maximum phonation exercises may compliment improvements in V̇O_2_peak in individuals with HF, and in turn, patient outcome [27]. Further research in a larger population cohort (including HFrEF and HFpEF) is required to fully understand whether it is only the marker itself improved, or a favourable improvement of underlying disease pathophysiology through maximum phonation exercises alone.

### Creak percent

Three studies assessed alterations in voice following diuretic treatment for decompensated HF, with the aims of exploring vocal biomarkers associated with disease progression [11, 18, 29]. Murton and colleagues consistently observed that creak percent ((number of creaky frames divided by the number of voiced frames) x 100) was higher following treatment in a pilot study and follow-up study assessing individuals with decompensated HF [18, 29]. This suggests that the vocal cord superior layer slackens following treatment due to reduced oedema, which in turn affects phonatory mechanics associated with creaky voice. The pilot study also reported a reduction in cepstral peak prominence on discharge. However, this finding was not replicated in the larger clinical study of 2023 [18, 29]. Using a different method to Murton and colleagues, another study assessed automated speech analysis and its ability to detect pulmonary oedema in acute decompensated HF [11].

### Pause ratio

Pause ratio is an additional vocal biomarker with promising clinical potential for monitoring HF disease progression [30, 45]. The measure was independently higher in acute decompensated HF when compared to stable chronic HF [30]. Importantly, differences in measurements were observed in individuals with low levels of oedema and/or dyspnoea, and independent of age, sex, and ejection fraction. Therefore, this biomarker may allow more timely therapeutic intervention of decompensation in HF than currently available [30].

### Speech speed, articulation, and sound intensity

Speech speed, articulation time, and sound intensity may be utilised to detect worsening HF [24, 30]. Decompensated HF is associated with a reduction in the speed of speech and articulation [30]. This, combined with the previously described increase in pause ratio, are consistently observed across speech tasks in individuals with progression of HF [30]. Individuals with acute congestive HF are also observed to have higher level of lung auscultation (i.e., presence of crackles or rales) compared to those with stable chronic HF [30]. Regarding sound intensity, this voice feature is significantly correlated with BNP levels. However, there is little description in publication of pathophysiological relation to HF [24].

Although the specific vocal biomarkers evaluated were not explicitly reported, some studies identified significant differences across five voice-derived measures when comparing recordings obtained at hospital admission and discharge [11]. These measures were selected based on characteristics including high temporal and spectral resolution, Euclidean distance, a symmetric nonlinear spectral ratio, nonlinear amplitude and frequency mapping, and autoregressive modelling of the voice spectrum. The observed changes highlighted the potential utility of vocal signal alterations in identifying pulmonary oedema and reflecting the clinical status of individuals with HF [11]. Such voice-based monitoring approaches may also prove valuable for assessing patient response to recent pharmacological adjustments or lifestyle interventions. This is particularly relevant given the hydration-sensitive nature of the vocal folds, whereby changes in fluid status may induce measurable alterations in vocal fold biomechanics and, consequently, in extracted voice features.

## Telemedicine and the future of voice in heart failure

From 2011 to 2023, the percentage of individuals in the UK that owned a smartphone doubled from 44% to 91% [46]. Notably, from 2012 to 2023 the greatest increase in smartphone users occurred in age groups 35–54 years (i.e., 43% to 99%), 55–64 years (i.e., 19% to 86%), and ≥ 65 years (i.e., 5% to 80%) [47]. These statistics pose promise for the use of telemedicine for monitoring disease progression and risk stratification of HF, a clinical syndrome with an age-associated prevalence [2, 48].

Across age groups, voice-based interfaces have been shown to be accessible and usable by older adults, as well as by individuals with lower literacy levels and/or cognitive impairments [49]. In individuals with HF, with a reported mean age of 55 years, engagement with telehealth interventions has been demonstrated to be feasible [49]. However, lower levels of engagement observed among individuals prescribed a greater number of HF medications (indicative of increased disease burden) suggest that existing interfaces may not adequately accommodate the needs of this subgroup, underscoring the need for further research to optimise interface design and personalisation [49].

Voice-based conversational agents used globally, including commercial platforms such as Amazon Alexa, have been employed as reference models for the development of clinical voice-enabled systems [49]. In parallel, routine clinical telephone interactions have been successfully leveraged as a medium for data collection within research contexts [31]. Voice command samples from individuals living with HF may represent a valuable source of data, particularly once machine learning models capable of extracting clinically relevant biomarkers from speech are sufficiently validated.

Nevertheless, the collection and use of voice data raise important ethical and legal considerations. Key issues include data ownership, governance, and consent, as well as the timing and conditions under which such data are collected and processed. Addressing these concerns will be essential to ensure the responsible integration of voice-based technologies into clinical research and practice.

Smartphone applications such as HearO (Cordio Medical) also show promise for preventive care by utilising speech technology to processes speech and detect minute alterations in text-dependent speaker verification [50]. The technology may determine changes in the clinical status in HF individuals (i.e., at admission; and at discharge) through the analysis of speech measures [11, 51]. The present digital age may therefore be beneficial for the passive collection of vocal data, improving adherence to remote care, and provision of new, clinically relevant biomarkers for monitoring and diagnosing HF.

## Challenges for vocal biomarkers in the clinical setting

Implementation of voice recognition techniques in routine practice is not without challenges. The first challenge is ensuring compliance with health data protection regulations due to associated privacy risk. For machine learning, large files of data are required to generate predictions. Voice data constitutes personal data and may also be analysed to infer additional personal characteristics, including sex, age, and ethnicity. Therefore, all data must be stored and processed in line with the EU guidelines for the usage of artificial intelligence and General Data Protection Regulation (GDPR) [52].

In addition to ensuring compliance, the reliability, sensitivity, and validity of all potential and defined biomarkers independent of language must be calculated. Furthermore, relating specific biomarkers to mechanisms directly associated with HF and not confounders or correlates like smoking and BMI is an important consideration. Regardless of strong reliability, sensitivity, and validity of such technology, the package must be accepted by the intended user (i.e., patients and healthcare professionals) to ensure smooth integration into the clinical care diagnostic pathway.

## Limitations of the evaluated literature

The reviewed body of research presents several important limitations. First, the existing screening studies are not conducted within the most clinically relevant domains and frequently rely on healthy controls as comparators, which may limit the applicability of findings to real-world diagnostic settings. Moreover, none of the studies examined longitudinal changes in the voices of individuals with stable HF over an extended period, such as two months. A pilot trial currently underway in Switzerland aims to address this gap by evaluating whether vocal biomarkers can augment standard care through comparison with daily weight and blood-pressure monitoring using a mobile device [52].

Although most studies excluded participants with known voice disorders, none implemented formal screening procedures to identify undiagnosed conditions. As a result, unrecognised voice pathology may have introduced bias into the datasets. Future research should incorporate systematic screening for voice disorders to improve data quality and interpretability.

Sample size represents another major limitation. Only two studies included more than 120 participants with HF [14, 31], which is insufficient for developing clinically robust algorithms or reliably identifying disease-specific vocal biomarkers. To overcome this constraint, larger multicentre studies are now emerging, including the European STRATIFY-HF study, which aims to recruit 1,600 individuals at risk of or living with HF [53]. This study incorporates language-independent voice features, 12-month follow-up, and machine-learning approaches, offering the potential for more generalisable findings.

Finally, voice-feature extraction is inherently sensitive to background noise. Although efforts were made to minimise environmental interference, its influence cannot be fully excluded, particularly in studies relying on remote, home-based monitoring [11, 51]. This remains an important methodological consideration for future research employing real-world voice data collection.

## Conclusion

Through the pathophysiological effects of HF on vocal function, voice analysis shows promise for facilitating diagnosis, predicting adverse outcome, and informing clinical management. Due to the ease of recording, and non-invasive nature of voice feature acquisition, it has the potential for future implementation in the clinical care pathway for HF. However, evidence supporting the risk-stratification and screening accuracy of vocal biomarkers remains limited, particularly in individuals with non-specific symptoms. Further research is needed to clarify the clinical and screening utility of voice features in HF.

## Data Availability

No datasets were generated or analysed during the current study.
